# Wolfberry prevented liver damage caused by anti-tuberculosis drugs associated with the YAP1/FXR pathway through gut microbiota

**DOI:** 10.3389/fmicb.2025.1700408

**Published:** 2025-11-24

**Authors:** Dan Wang, Yajun Xiong, Zhihan Liu, Xiaoyong Song, Jiaojiao Liu, Yanli Gong, Zhuanzhuan Li, Xinli Shi

**Affiliations:** Laboratory of Integrated Medicine Tumor Immunology, Shanxi University of Chinese Medicine, Taiyuan, China

**Keywords:** wolfberry (*Lycium barbarum* L.), antituberculosis drug-induced liver injury, intestinal flora, intestinal barrier function, FXR, CYP7A1, YAP1

## Abstract

**Background:**

The prevalence of anti-tuberculosis drug-induced liver injury (AT-DILI) is significant, with severe cases potentially leading to liver failure or mortality. Research indicates that first-line anti-tuberculosis drugs (ATDs), including rifampicin (RIF) and isoniazid (INH), cause a lasting disruption of gut flora, which is significantly associated with drug-induced toxicity. Wolfberry (*Lycium barbarum* L.) is frequently utilized in traditional Chinese medicine for the treatment of hepatic and renal disorders. The mechanism by which wolfberry prevents AT-DILI remains unclear. This work aimed to explore how wolfberry prevents AT-DILI by modulating the composition and functionality of intestinal microbiota and enhancing intestinal barrier integrity, hence elucidating its protective mechanism via the gut-liver axis.

**Methods:**

Forty male Kunming (KM)mice were randomly allocated into four groups: normal, model, wolfberry and Polyenylphosphatidylcholine (PPC) group (*n* = 10/group). The normal group received ultrapure water via gavage daily, whereas the other three groups were administered ultrapure water, wolfberry decoction, and PPC via gavage three hours prior to RIF and INH daily for 21 days. Twenty additional mice were made pseudo-germ-free through a one-week oral injection of antibiotic (ATB) water, subsequently categorized into ATB and ATB + wolfberry groups. The remaining intervention strategies were identical to those previously mentioned. Subsequently, serum Alanine Aminotransferase (ALT) and Aspartate Aminotransferase (AST), serum and tissue Total Bile Acid (TBA) levels, and serum creatinine (CRE) levels were assessed. Intestinal contents were obtained for 16S rRNA sequencing, and pathological investigation was conducted on liver, kidney, and intestinal tissues. The expression levels of Yes-associated protein 1 (YAP1), Farnesoid X Receptor (FXR), and Cytochrome P450 Family 7 Subfamily A Member 1 (CYP7A1) in hepatic tissue were assessed.

**Results:**

Results showed AT-DILI decreased beneficial gut microbiota abundance and increased CYP7A1 expression associated with the YAP1/FXR pathway. Wolfberry intervention enriched beneficial microbiota, increased goblet cells, upregulated tight junction protein ZO-1, and enhanced intestinal barrier function, while reducing serum ALT, AST, and TBA. Additionally, wolfberry increased nuclear YAP1 expression, activated FXR, and downregulated CYP7A1 to reduce TBA synthesis. The key finding is that after antibiotics clear the gut microbiota, wolfberry failed to activate the YAP1/FXR pathway.

**Conclusion:**

Wolfberry comprehensively prevented liver damage under the condition of gut microbiota presence by enhancing gut microbiota diversity, strengthening intestinal barrier function, associating with the YAP1/FXR pathway.

## 1 Introduction

The 2023 Global Tuberculosis Report indicates that tuberculosis (TB) resulted in 1.3 million fatalities globally in 2022, maintaining its status as the second-highest cause of death from a single infectious agent following COVID-19. China is third in estimated tuberculosis incidence among the 30 nations with a high burden of the disease, following Indonesia and India (World Health O, 2023). Rifampicin (RIF) and isoniazid (INH) are the primary antituberculosis medications. Nevertheless, their administration often results in side effects, with hepatotoxicity being the most significant issue (Song et al., 2023). Thus, an AT-DILI has become the primary adverse consequence of short-course tuberculosis chemotherapy. Recent years have witnessed minimal advancement in anti-TB drug development, highlighting an urgent necessity for innovative therapeutics.

Asian countries demonstrate elevated tuberculosis incidence rates relative to Western nations, resulting in the notable prevalence of AT-DILI in these areas. In China, AT-DILI constitutes 22.0–31.3% of reported drug-induced liver injury (DILI) cases, rendering it the predominant DILI etiology in Asia and a significant factor in acute liver failure (ALF) and acute-on-chronic liver failure (ACLF) (Guo et al., 2010). First-line antituberculosis drugs, including isoniazid, rifampicin, and pyrazinamide, demonstrate significant hepatorenal damage (Song et al., 2023). Currently, therapy alternatives for DILI are constrained, requiring novel therapies.

Yes-associated protein 1 (YAP1), a crucial regulator of the Hippo pathway (Grijalva et al., 2014), significantly influences liver pathology by regulating hepatocyte survival and regeneration (Mohseni et al., 2019). Wolfberry mitigates DILI by increasing the amount of *Akkermansia muciniphila* in the gastrointestinal tract and upregulating hepatic YAP1 expression, therefore strengthening liver function (Liu et al., 2023). The exact method by which wolfberry influences YAP1 to mitigate AT-DILI remains inadequately elucidated.

The farnesoid X receptor (FXR)/cholesterol 7α-hydroxylase (CYP7A1) axis functions as the principal regulatory mechanism for bile acid metabolism, with its dysregulation leading to bile acid buildup and worsening liver injury (Li et al., 2021). FXR, a key regulator of bile acid homeostasis, inhibits CYP7A1 expression upon activation (Song et al., 2025). CYP7A1, a liver-specific cytochrome P450 enzyme, facilitates the rate-limiting transformation of cholesterol into 7α-hydroxycholesterol during bile acid production (Rizzolo et al., 2021). The gut microbiota significantly influences bile acid biotransformation, while bile acids, in turn, affect microbial makeup. The metabolic interaction between bacteria and the host underscores the therapeutic potential of targeting gut-liver axis signaling.

Wolfberry (*Lycium barbarum* L.), a geographically specific herb predominantly cultivated in Ningxia (China). It has been used for millennia in traditional Chinese medicine, with its dried ripe fruits as the exclusive medicinal portion, which has the effect of nourishing the liver and kidney (Commission CP, 2020). The effective components of wolfberry or its compound preparations are widely used in the treatment of liver and kidney diseases in clinical practice (Gao et al., 2021; Li et al., 2013). Recent research has shown that wolfberry possesses preventive properties against diverse types of drug-induced oxidative stress and inflammation (Liu et al., 2023; Nie et al., 2025; Zhao et al., 2025). Wolfberry contains a variety of bioactive compounds, such as *L. barbarum polysaccharides*, flavonoids, and *betaine*, which are believed to enhance immune function and protect tissues from damage (Rjeibi et al., 2018; Ma et al., 2025). However, its specific role in the protection against liver and kidney injury induced by Anti-tuberculosis drugs (ATDs) remains to be fully elucidated.

Consequently, our research focuses on the modulation of intestinal flora by Wolfberry, associated with the YAP1/FXR pathway, the reduction of total bile acids, and the prevention of AT-DILI.

## 2 Methods

### 2.1 RIF + INH-induced DILI model

In 1944, Professor Tang Feifan introduced Swiss mice from the Hofkine Institute in India and raised them at the Central Epidemic Prevention Office in Kunming, hence the name Kunming mice. In 2011, SPF (Beizhongg) Biotechnology Co., Ltd. introduced and cultivated the mice from the National Rodent Laboratory Animal Seed Center. Five-week-old male Kunming (KM) Male mice, weighing about 20 g (SiPeiFu, Beijing, China) were bred in SPF environment. In a 12-h cycle of darkness, animals had free access to water and food. This experiment was approved by the Animal Ethics Committee of Shanxi University of Traditional Chinese Medicine (AWE20240434).

Construction of a mouse model of DILI caused by antituberculosis drugs: Using rifampicin suspension and isoniazid suspension, mice were gavaged continuously for 21 days (Lin et al., 2025) (isoniazid 50 mg/kg + rifampicin 100 mg/kg (World Health O, World Health O, 2010; Song et al., 2025; Bai et al., 2022), the suspension was formulated with sodium carboxymethylcellulose at a mass concentration of 0.5%), The model was successfully constructed. Mice in the Wolfberry group were given Wolfberry decoction (0.3 g/mL, 0.1 mL/pupil), and mice in the PPC group were given PPC (0.053 g/mL PPC, 0.1 mL/pupil). After 3 h, mice in the three groups, except for the Normal group, were given INH50mg/kg + RIF100mg/kg. After 21 days of intervention, the mice were sacrificed and the liver, kidney, blood and intestinal tissues of the mice were collected.

### 2.2 Alanine aminotransferase (ALT) and aspartate aminotransferase (AST)

The assay was performed according to the instructions of the kit (C010-2-1 and C009-2-1, njjcbio, Nanjing, China). OD (Optical Density) values were measured at a wavelength of 510 nm using a multifunctional microplate reader (SpectraMax Mini, Molecular Devices).

### 2.3 Determination of total bile acids (TBA) content

The assay was performed according to the instructions of the kit (AII0-1-1, njjcbio, Nanjing, China). The OD values of the samples were read at 405 nm using a multifunctional microplate reader (SpectraMax Mini, Molecular Devices).

### 2.4 Determination of creatinine (CRE) content

The assay was performed according to the instructions of the kit (C011-2-1, njjcbio, Nanjing, China). The OD values of the samples were read at 546 nm using a multifunctional microplate reader (SpectraMax Mini, Molecular Devices).

### 2.5 Hematoxylin and eosin (H&E) staining

The liver, gut, and kidney of mice were processed into paraffin blocks. The tissues were subsequently stained with H&E (DH0020, LEAGENE) to assess the histopathological morphology, and images were captured using a microscope (DM4B, Leica, Germany).

### 2.6 16S rRNA sequencing

Fecal samples from mice were collected and sent to Shanghai Personalbio Technology Co., Ltd. for bacterial diversity analysis using the Illumina NovaSeq 6,000 platforms targeting the V3-V4 region. The primer sequences used were (F: ACTCCTACGGGAGGCAGCA; R: GGACTACHVGGGTWTCTAAT) Through processes of merging, filtering, and denoising of the raw data, valuable data were obtained. The specific composition of each sample was displayed at different taxonomic levels.

### 2.7 Periodic acid-Schiff staining (PAS)

Goblet cells of mice colon and ileum were stained using PAS kit (R20526, Yuanye, Shanghai, China). Utilizing a microscope (DM2500, Leica Camera, Germany) for observation and image acquisition.

### 2.8 Western blot

Primary antibodies were FXR (TA809306S, OriGene, 1:1000), CYP7A1 (DF2612, Affinity Biosciences, 1:1000), YAP1 (14074S, CST, 1:1000), and GAPDH (AB0037, Abways, 1:5000). HRP-linked secondary antibodies were goat anti-rabbit IgG (abs20040, Absin; 1:5000) and goat anti-mouse IgG (H + L) (AB0102, Abways; 1:5000).

### 2.9 Immunohistochemistry (IHC)

Liver and colon tissue sections underwent high-pressure antigen retrieval prior to immunohistochemical processing. Subsequent steps followed the PV-9000 IHC kit protocol (ZSGB-BIO, Beijing, China). Primary antibody incubations employed were anti-FXR (TA809306S, ORI-GENE; 1:150), anti-CYP7A1 (DF2612, Affinity Biosciences; 1:100), anti-YAP (D8H1X) XP (14074S, CST; 1:200). Tissue sections were visualized utilizing a microscope (DM4B, Leica, Germany). Anti-ZO-1 tight junction protein antibodies [BLR092G] (ab276131, 3EDC Abcam, dilution 1:300). The sections are imaged using.

### 2.10 Statistical analysis

Maps and statistical analysis were done with GraphPad Prism 9.5. One-way ANOVA was employed to contrast groups of mice, and then Tukey’s test was used to make two-by-two comparisons. A *p*-value of less than 0.05 was considered statistically significant.

## 3 Results

### 3.1 Wolfberry prevented liver damage in mice induced by anti-tuberculosis drugs

**Figure 1:**
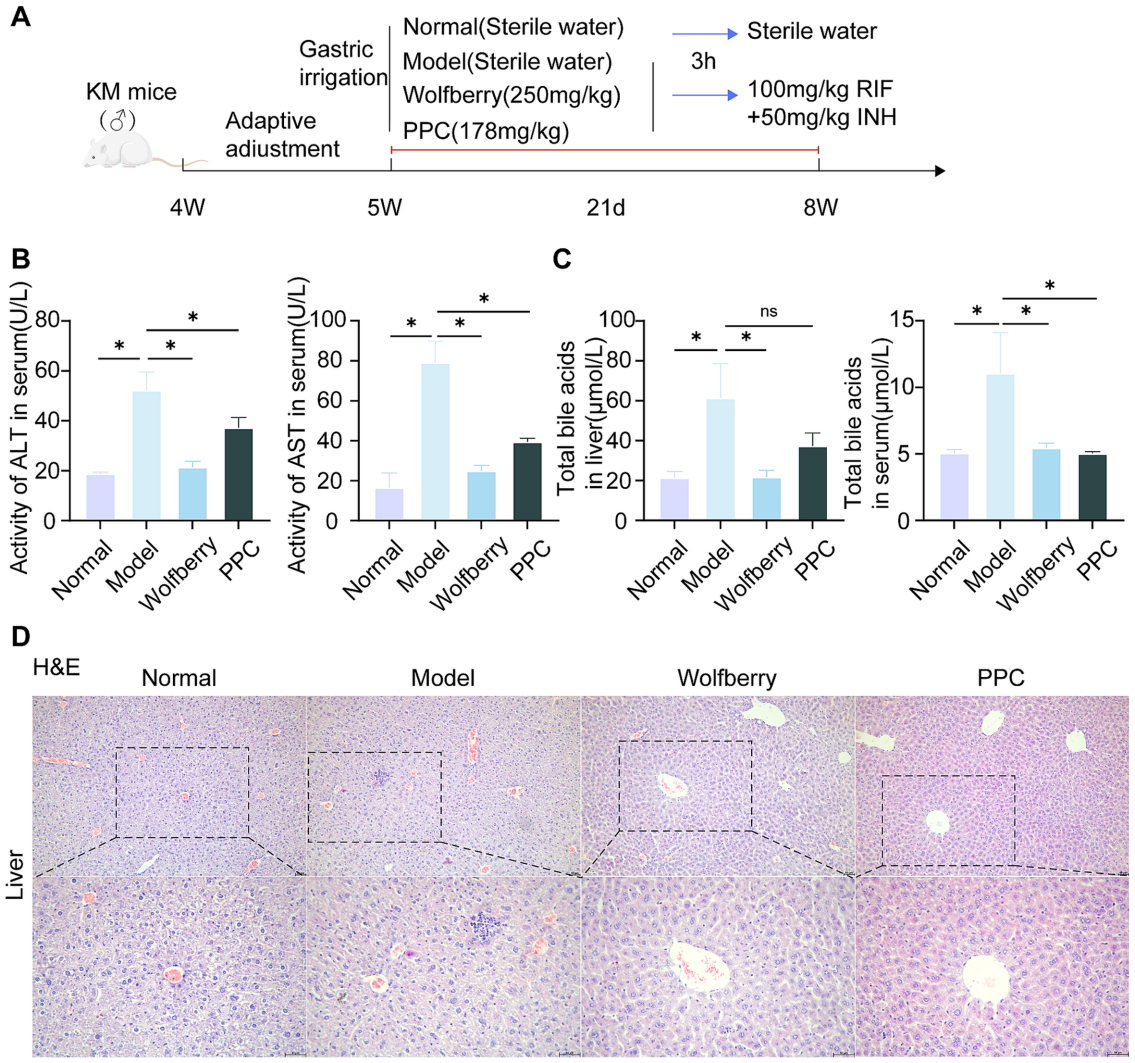
Wolfberry prevents liver damage in mice induced by anti-tuberculosis drugs. **(A)** Chematic diagram of RIF + INH-induced AT-DILI mouse model. **(B)** ALT and AST activities of mice in each group. **(C)** Hepatocyte morphology in H&E-stained liver sections (scale bar: 50 μm). **(D)** TBA levels in serum. ^*^p < 0.05.

The group conducted pre-tests, including H&E staining of liver tissue, serum alanine aminotransferase ALT, AST, and TBA assessments to evaluate the efficacy of Wolfberry (Figure 1). Anti-tuberculosis drugs (ATDs) significantly elevated 52.22 ± 7.32 U/L for ALT and 79.12 ± 10.70 U/L for AST. While the content of ALT is 18.82 ± 0.59 U/L, and AST is 16.62 ± 7.30 U/L in serum from the normal group. This represented a 2.77-fold increase in ALT and a 4.76-fold increase in AST compared to normal levels (Figure 1B). Further, characteristic streak-like alterations, inflammatory infiltration, and punctate necrotic foci were observed in liver tissues by H&E analysis (Figure 1D). Wolfberry inhibited the contents of ALT (21.50 ± 2.23 U/L) and AST (24.98 ± 2.70 U/L) in serum (Figure 1B). Moreover, it reduced focal necrosis and inflammatory infiltration (Figure 1D), suggesting a preventive effect of wolfberry against AT-DILI. Critically, the wolfberry intervention restored hepatic function to near-physiological states. Achieved ALT and AST values showed no statistically significant difference from the normal baseline, indicating complete functional restoration (Figure 1B). PPC is a pharmacological agent utilized for the clinical care of AT-DILI and serves as a positive control in this investigation. PPC reduced the liver biochemical indicators and decreased the infiltration of inflammatory cells (Figures 1B–D).

The liver is the sole organ responsible for bile acid production (Jia et al., 2024). The buildup of TBA was measured in hepatic tissue and systemic circulation to evaluate cholestatic damage. The intervention of ATDs resulted in an elevation in TBA. Wolfberry decreased tissue TBA to 21.77 ± 3.23 μmol/L and serum TBA levels to 5.46 ± 0.37 μmol/L, indicating decreases of 2.82-fold and 2.07-fold, respectively. The wolfberry treatment markedly diminishes the aberrant buildup of bile acids (Figure 1C). The effectiveness of the positive control medication PPC is variable, with TBA levels measuring 5.04 ± 0.13 μmol/L in serum, approaching normal levels. The tissue accumulation level rose to 37.37 ± 6.54 μmol/L and failed be reverted to normal levels (Figure 1C). In stark contrast, wolfberry efficiently reduced abnormal TBA buildup in both tissue and circulatory compartments to normal levels. These data demonstrated that AT-DILI induced systemic elevation of TBA. Critically, whereas PPC normalized serum TBA levels, it exerted no significant effect on tissue TBA accumulation. In marked contrast, wolfberry effectively prevented pathological TBA elevation in both compartments.

### 3.2 Wolfberry balanced gut microbiota

Bile acids (Bas), essential gut microbiota metabolites, undergo enterohepatic recirculation with 95% reabsorbed before the terminal ileum (Collins et al., 2023). Previous research indicates that wolfberry modifies the composition of gut microbiota (Liu et al., 2023), necessitating 16S rRNA sequencing of murine fecal samples.

**Figure 2:**
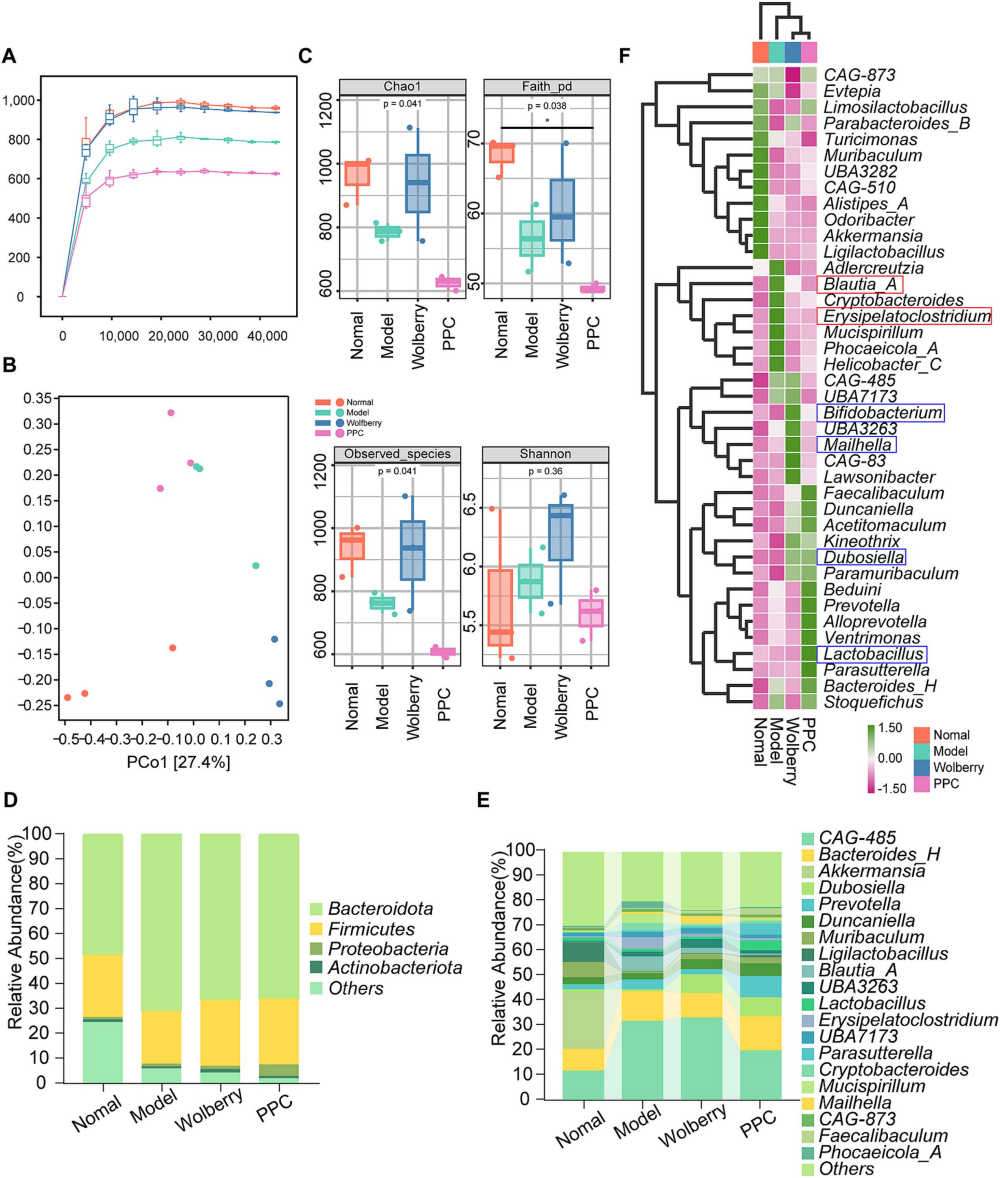
Wolfberry balanced gut microbiota. **(A)** Sparse curves of 16S rRNA gene sequencing in different groups of mice. **(B)** PCoA plots of mice in different groups. **(C)** Alpha diversity indices in different groups of mice. (Chao1, Faith_pd, Observed species, Shannon,). **(D,E)** Microbiota composition at the phylum level (top 10) and flora composition at the genus level (top 20) for each group of mice. **(F)** Heat map showing the top 20 species at the genus level in each group of mice.

Rarefaction curves demonstrated adequate sequencing depth, as the plateauing of the curves validated data sufficiency and reliability (Figure 2A). Following the intervention with ATDs, microbial diversity diminished, but the intake of wolfberry markedly reinstated microbial diversity to nearly normal levels. The variety within the PPC group is minimal (Figure 2A). Principal coordinate analysis (PCoA) demonstrated clear clustering among the four groups, highlighting significant compositional disparities (Figure 2B).

The alpha diversity indices (Chao1, Faith-pd, Observed species) indicated greater microbial richness in the wolfberry group and the lowest diversity in the PPC group (Figure 2C). These data indicate that wolfberry mitigates reductions in microbial diversity and structural dysbiosis generated by AT-DILI.

Analysis of taxonomic makeup revealed alterations at both the phylum and genus levels. The Firmicutes/Bacteroidetes (F/B) ratio, an essential marker of gut homeostasis, was maximal in the Normal group and minimal in the Model group (Figure 2D). Decreased F/B ratios, resulting from Firmicutes depletion or Bacteroidetes dominance, are linked to chronic inflammation and gastrointestinal problems. At the genus level, *Alistipes_A, Qdoribacter, Turicimonas, Muribaculum, CAG-510, Ligilactobacillus,* and *Akkermansia* exhibited the highest abundance in the Normal category. Wolfberry intervention showed an enrichment of *CAG-485, Bifidobacterium, Mailhella,* and *Lawsonibacter,* whereas the PPC group revealed elevated levels of *Lactobacillus, Parasutterella,* and *Bacteroides_H* (Figures 2E,F).

### 3.3 Wolfberry improves the intestinal barrier function

**Figure 3:**
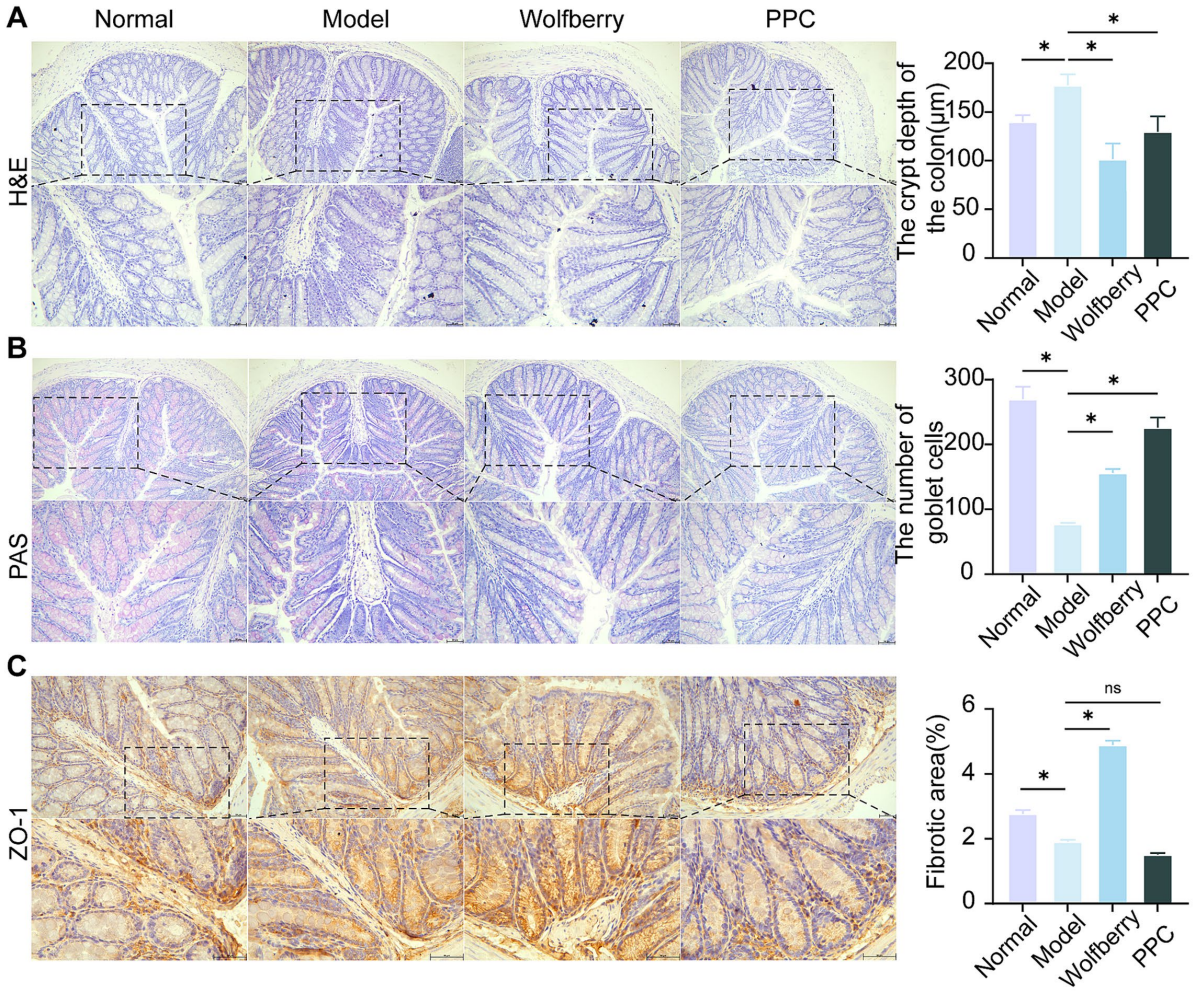
Wolfberry improves the intestinal barrier function. **(A)** H&E staining to observe the pathological morphology of the colon and counting the depth of crypts (scale bar, 50 μm). **(B)** Colonic PAS staining and counting of the number of cup cells (scale bar, 50 μm, cup cells in pink color). **(C)** The positive expressions of ZO-11 in the mice liver were detected by IHC and quantification of its expression intensity by measuring the gray value. (scale: 50 μm). ^*^p < 0.05.

The colon, a principal site for gut microbiota colonization (Tropini et al., 2017), was examined using H&E and PAS staining to assess intestinal barrier integrity in light of the noted changes in serum TBA levels and microbial composition among treatment groups. The intestinal tissue exhibited considerable structural damage, along with pronounced infiltration of inflammatory cells, as evidenced by H&E staining following tuberculosis medication intervention. In contrast, the wolfberry intervention preserved crypt depth and mitigated mucosal injury (Figure 3A).

PAS staining revealed the distribution and abundance of goblet cells, which primarily synthesize and secrete mucins to form a protective mucosal barrier for epithelial cells (Johansson et al., 2011). The substantial reduction in goblet cell numbers following the introduction of ATDs indicates that ATDs inflicted harm on the intestinal mucosa. Conversely, the amount of goblet cells was significantly increased by wolfberry intervention, demonstrating its role in intestinal mucosa regeneration. The positive control medication PPC produced an increase in goblet cell quantity (Figure 3B).

Tight junctions, especially Zonula Occludens-1 (ZO-1), was essential for preserving selective permeability and facilitating mucosal healing (Kuo et al., 2021). ZO-1 is uniformly distributed within the intestinal epithelium of healthy mice. Conversely, following intervention of ATDs, ZO-1 expression was reduced, and its distribution was confined to the crypt areas. The enhancement of ZO-1 expression following wolfberry intervention significant advancement in mucosal repair (Figure 3C). These findings combined suggested that wolfberry improves intestinal barrier function in AT-DILI.

### 3.4 Wolfberry prevents liver damage induced by anti-tuberculosis drugs through intestinal flora

**Figure 4:**
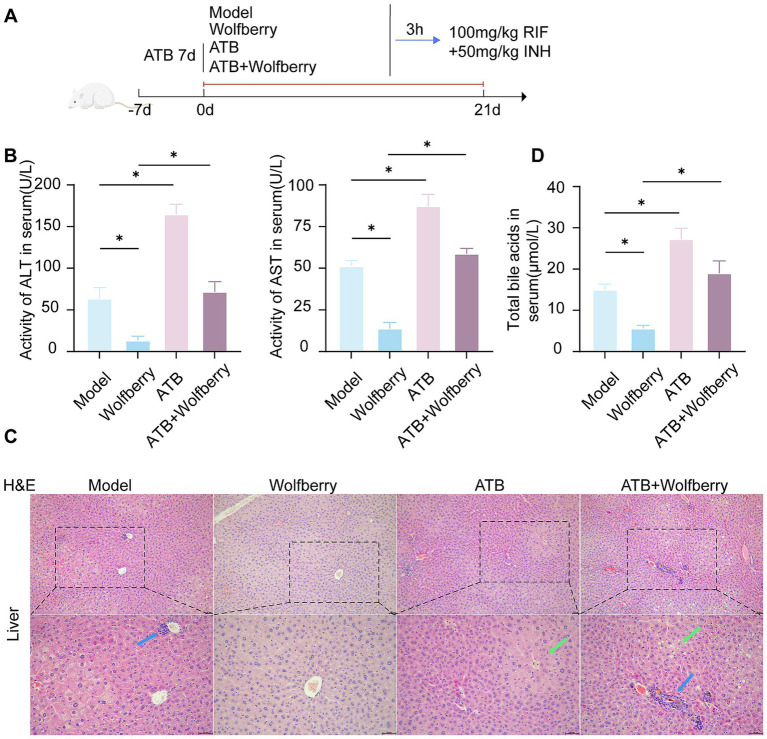
Wolfberry prevents liver damage induced by anti-tuberculosis drugs through intestinal flora. **(A)** Schematic diagram of AT-DILI mouse model after ATB water intervention. **(B)** ALT and AST activities of mice in each group. **(C)** Hepatocyte morphology in H&E-stained liver sections (Blue arrows indicate inflammatory infiltration, green arrows indicate ballooning degeneration of hepatocytes. Scale bar: 50 μm). **(D)** TBA levels in serum. * *p* < 0.05.

**Figure 5:**
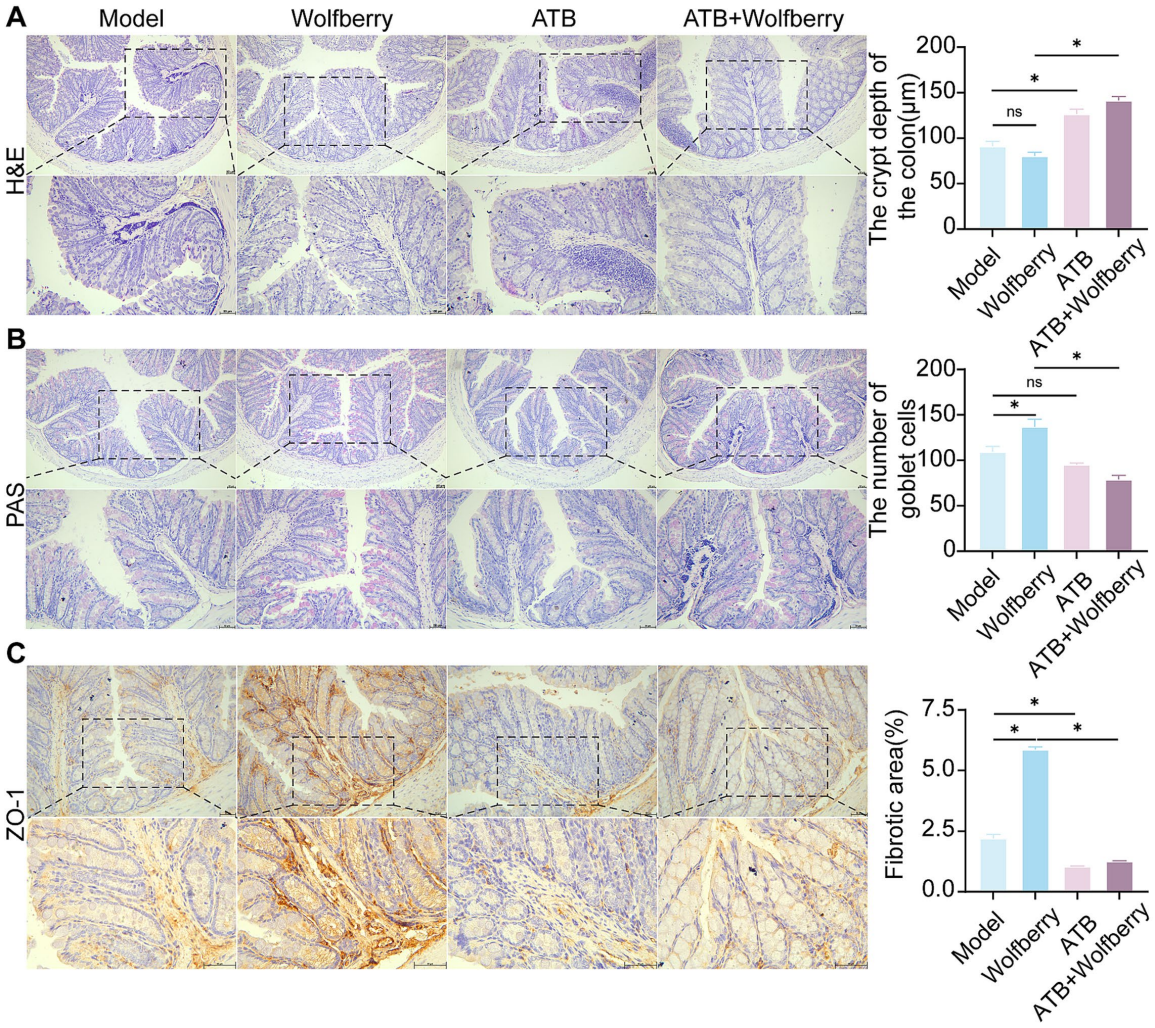
Health benefit of wolfberry through intestinal flora. **(A)** H&E staining to observe the pathological morphology of the colon and counting the depth of crypts (scale bar, 50 μm). **(B)** Colonic PAS staining and counting of the number of cup cells (scale bar, 50 μm, cup cells in pink color). **(C)** The positive expressions of ZO-1 in the mice liver were detected by IHC and quantification of its expression intensity by measuring the gray value. (scale: 50 μm). ^*^p < 0.05.

To ascertain if the hepatoprotective action of wolfberry against AT-DILI is facilitated through intestinal flora, intestinal flora was depleted using antibiotic-containing (ATB) water (Figure 4A). After ATB water intervention, the ALT and AST values increased to 2.58 times and 1.70 times, respectively, compared to SPF mice treated with ATDs (Figure 4B). Furthermore, there was an aggravation of liver tissue pathology (Figure 5C), substantiating that the eradication of the microbiota intensified AT-DILI damage. Normalization of ALT and AST levels was achieved with wolfberry intervention. Subsequent to the elimination of the gut flora, elevated 71.87 ± 12.12 U/L for ALT and 58.78 ± 3.17 U/L for AST (Figure 4B), with increased inflammatory infiltration in the liver tissue (Figure 4C). This suggested that wolfberry provided a protective effect through the gut flora. After the removal of the gut microbiota, severe ballooning degeneration and inflammatory cell infiltration, as evidenced by H&E staining (Figure 4C). Wolfberry alleviated liver pathological damage through gut microbiota. Collectively, these results showed that gut microbiota ablation exacerbates hepatotoxicity and wolfberry alleviated liver pathological damage through gut microbiota.

### 3.5 Health benefit of wolfberry through intestinal flora

ATB water intervention damaged structural and increased the inflammatory cell infiltration in gut (Figure 5A). Wolfberry repaired the intestinal damage in SPF mice, while did not in ATB-treated mice during ATDs treatment. These results showed that wolfberry requires gut microbiota to repair the inflammatory infiltration in the intestinal barrier.

The quantity of goblet cells diminished following the ATB water intervention, as quantitatively demonstrated by PAS staining (Figure 5B). Wolfberry intervention increased the number of goblet cells, but this effect disappeared after the removal of the gut microbiota. This suggested that wolfberry particularly increased the quantity of goblet cells through gut microbiota.

After the ATB water intervention, the expression of ZO-1 was downregulated (Figure 5C). Only wolfberry upregulated ZO-1 expression in the presence of gut microbiota. These findings suggested that wolfberry enhanced ZO-1 expression through the mediation of gut microbiota. Overall, wolfberry strengthened intestinal barrier function by relying on gut microbiota to suppress inflammation, increase goblet cells, and enhance the expression of tight junction proteins.

### 3.6 Wolfberry prevents kidney damage through intestinal flora

As relevant studies indicated that antituberculosis drugs cause renal injury in mice (Lin et al., 2025). An increase in CRE concentration is an important indicator of kidney function damage (KDIGO, 2024). After the intervention with ATDs, creatinine increased by 3.87 times, accompanied by edema of the renal tubular epithelial cells and inflammatory infiltration. This suggested that ATDs led to kidney damage.

**Figure 6:**
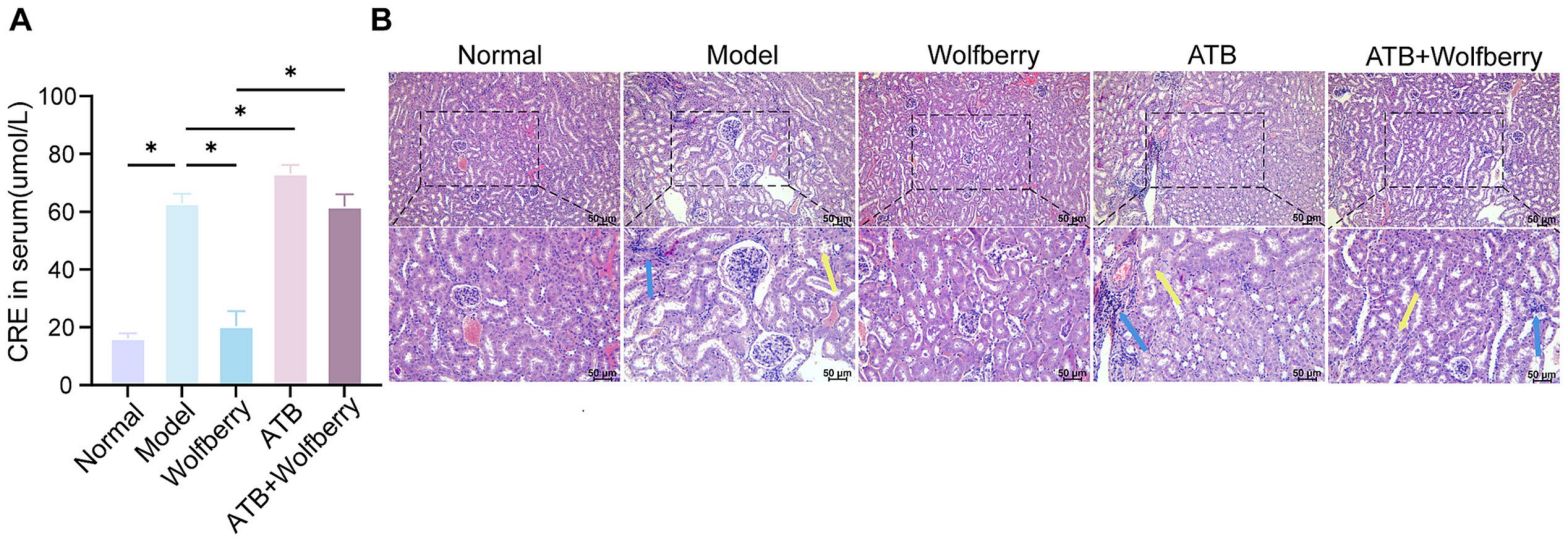
Wolfberry prevents kidney damage through intestinal flora. **(A)** Cre levels in each group of mice. **(B)** Morphology of glomeruli and tubules in H&E-stained renal tissue sections (The blue arrows indicate inflammatory infiltration, while the yellow arrows indicate edema of the renal tubular epithelial cells, cell swelling, loose cytoplasm, and pale staining. Scale bar, 50 μm).

Wolfberry inhibited the contents of CRE (20.47 ± 5.12 μmol/L) in serum, which was a 3.07-fold reduction compared to SPF mice treated with ATDs, and it alleviated tissue pathological damage, suggesting that wolfberry had a protective effect on the kidneys against AT-DILI. After ATB water intervention, the damage worsened with CRE levels being 1.16 times higher than in ATDs-treated mice. There was an increase in cellular edema and inflammatory infiltration, indicating that the depletion of the microbiome exacerbated kidney injury (Figures 6A,B). In summary, wolfberry prevented kidney damage induced by ATDs through the gut microbiota.

### 3.7 Wolfberry inhibited the formation of TBA associated with the YAP1/FXR pathway

**Figure 7:**
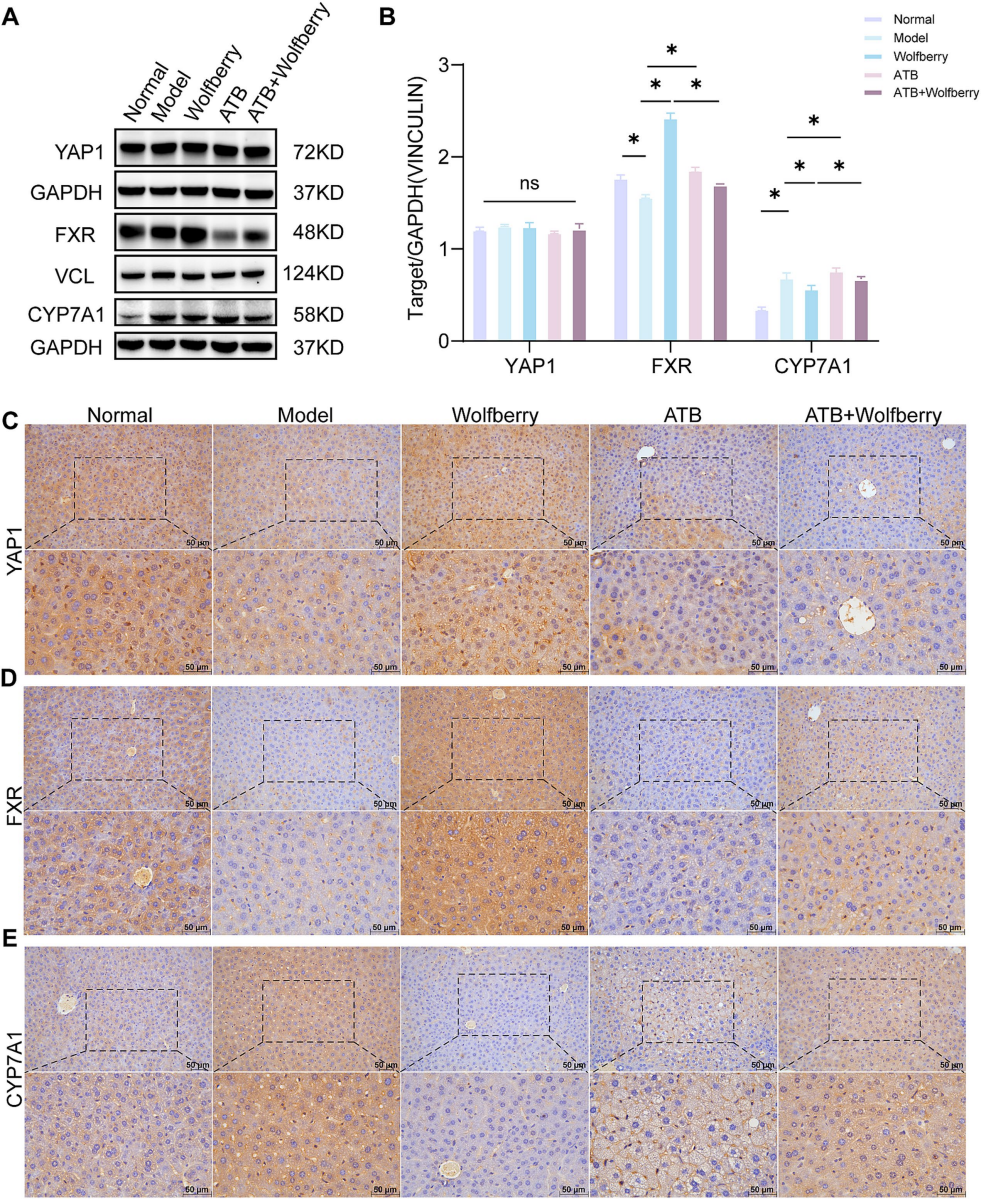
Wolfberry inhibited the formation of TBA associated with the YAP1/FXR pathway. **(A,B)** Western blotting was used to detect the expression of YAP1, FXR and CYP7A1 in the liver of mice with AT-DILI. GAPDH and VCL was used as the internal control. **(C–E)** The positive expressions of YAP1, FXR and CYP7A1 in the mice liver were detected by IHC (scale: 50 μm). *P < 0.5.

YAP1 played a crucial role in liver injury (Grijalva et al., 2014). Previous studies confirmed that wolfberry improves DILI by enhancing YAP1 expression in hepatocytes (Liu et al., 2023; Lu et al., 2024). Later studies used ATB water to examine the capacity of wolfberry to increase liver YAP1 expression through modulation of the gut flora. The findings reveal that total YAP1 levels were relatively stable. However, ATDs and ATB water treatment downregulate YAP1 expression, while it is upregulated in the liver cell nucleus from wolfberry (Figures 7A–C).

TBA accumulation in serum and tissues was significantly reduced following wolfberry intervention (Figure 1C). Concurrently, the administration of wolfberry increased the abundance of beneficial gut bacteria, as revealed by 16S rRNA sequencing (Figure 2F) and augmented the number of goblet cells and ZO-1 expression in the colon (Figures 3B,C). FXR served as a fundamental regulatory element in bile acid metabolism (Makishima et al., 1999). After the intervention with ATDs, the downregulation of FXR expression suggested a disruption of the bile acid negative feedback loop. The expression of FXR was markedly elevated following the wolfberry intervention, suggesting that the organism was compensatorily activating the FXR pathway to mitigate liver-intestinal damage resulting from bile acid accumulation or metabolic dysregulation. After using ATB water to eliminate the gut microbiota, the expression of FXR was downregulated following intervention with wolfberry (Figures 7A,B,D). This suggested that wolfberry activated FXR expression through the gut microbiota.

CYP7A1, a liver-specific cytochrome P450 enzyme critical for bile acid synthesis (Rizzolo et al., 2021), catalyzes cholesterol conversion to 7α-hydroxycholesterol as the rate-limiting step (Song et al., 2025). The activation of FXR inhibited the expression of CYP7A1. After intervention with ATDs, the expression of CYP7A1 was upregulated, showing a disruption of the bile acid negative feedback system, impaired FXR signaling, and increased toxicity due to TBA accumulation. The expression of CYP7A1 was downregulated following intervention with wolfberry, suggesting that wolfberry alleviates metabolic stress by activating FXR and inhibiting bile acid production (Figures 7A,B,E). In summary, wolfberry inhibited CYP7A1 by augmenting the expression of FXR, which efficiently decreased bile acid production. Studies showed that YAP1 positively regulated FXR expression in the liver, thereby inhibiting CYP7A1 (Lu et al., 2024). Collectively, wolfberry prevented AT-DILI associated with the YAP1/FXR pathway to suppress CYP7A1 and reduce bile acid synthesis.

## 4 Discussion

In Asian nations, the prolonged administration of first-line ATDs RIF and INH was the primary cause of intrinsic AT-DILI (Sharma et al., 2019; Branch, Chinese Medical Association Tuberculosis, 2024; Liu et al., 2020). In extreme instances, it may result in hepatic failure or mortality (Lu et al., 2023; Gijbels et al., 2019; Qi et al., 2020). Previous research has demonstrated that wolfberry increases the prevalence of *A. muciniphila* and the YAP1 expression to promote DILI (Liu et al., 2023). According to the findings we got, wolfberry comprehensively prevented liver damage under the condition of gut microbiota presence by enhancing gut microbiota diversity, strengthening intestinal barrier function, associating with the YAP1/FXR pathway.

This study demonstrated that wolfberry significantly decreased ALT and AST levels in AT-DILI mice, providing hepatorenal protective effects similar to those observed in the Normal group. Through 16S rRNA sequencing, we confirmed that wolfberry alters gut microbiota, enhances microbial diversity, and strengthens barrier function to alleviate hepatic metabolic stress (Gao et al., 2022). Despite the absence of clinical guidelines including wolfberry in standard AT-DILI prophylaxis, its multi-target hepatoprotective properties underscore its rising therapeutic significance. Comprehensive preclinical and clinical research substantiated the therapeutic effectiveness of wolfberry in liver illnesses, through the modification of gut microbiota (Lu et al., 2024; Lu et al., 2023; Duan et al., 2024). 16S rRNA sequencing revealed an increase in bacterial abundance in mice administered wolfberry. Wolfberry augmented *Dubosiella* and *Mailhella*, which are associated with anti-inflammatory and anti-aging benefits through immunological enhancement and restoration of the mucosal barrier (Zhang et al., 2024; Liu et al., 2023). Wolfberry significantly increased goblet cell quantities, crypt depth, and ZO-1 expression, consequently enhancing intestinal barrier integrity to prevent the transfer of hepatotoxic substances (Lu et al., 2023). However, concurrent used of antimicrobial water compromised the intestinal barrier and diminished the hepatoprotective effects of Wolfberry. Wolfberry enhanced intestinal barrier function and improved microbial diversity to alleviate liver damage.

The gut-liver axis emphasized the interaction of the gastrointestinal tract, liver, and microbiota, whereby the portal vein facilitates the flow of gut-derived substances that influence hepatic metabolic and immunological functions (Albillos et al., 2020). Research suggested that gut dysbiosis was significantly associated with the development of liver disorders (Szabo, 2015; Guan et al., 2022). The gut-kidney axis denoted the reciprocal physiological interaction between the gut and kidneys, principally facilitated by metabolic interdependencies and immunological pathways (Chen et al., 2019). Gut microbiota-derived compounds, such as short-chain fatty acids and uremic toxins, influenced renal function through systemic circulation, whereas kidney disorders concurrently modified the composition and activity of gut microbiota (Felizardo et al., 2016; Mariño, 2016). The findings of our study indicated that wolfberry contributes to the protection of the liver and kidneys, mitigating damage induced by antituberculosis medications via the enterohepatic and entero-renal pathways. Nonetheless, the investigation into whether the compounds in wolfberry or the secondary metabolites generated by gut flora contribute is the focus of our subsequent research.

Wolfberry regulated gut microbiota composition, augmented microbial diversity, and fortified intestinal barrier integrity. Moreover, it decreased TBA concentrations. Administering ATB water negated the therapeutic effects of wolfberry in AT-DILI mice. Bile acid metabolism predominantly transpired through the traditional pathway facilitated by CYP7A1 and the alternative pathway facilitated by CYP27A1, both governed by the FXR (Kullak-Ublick et al., 2004). In RIF + INH-induced AT-DILI mice, serum TBA levels were increased, hepatic FXR expression was markedly diminished, and CYP7A1 expression was raised. Wolfberry increased FXR expression and decreased CYP7A1 expression. The decreased regulation of CYP7A1 was crucial for the recovery of the liver to mitigate hepatotoxicity caused by increased bile acids (Huang et al., 2006), consistent with what we found. Furthermore, the regulatory impact of wolfberry on FXR was reduced when the gut microbiota was dysbiotic. Collectively, the results above collectively indicated that the advantageous impacts of wolfberry in enhancing FXR, suppressing CYP7A1, and ameliorating AT-DILI were realized through the modification of intestinal microbiota.

YAP1 was essential in liver injury and regeneration (Grijalva et al., 2014; Wang et al., 2022). Previous studies has shown that wolfberry increases nuclear expression of YAP1 in the hepatocytes of mice with APAP-induced DILI (Liu et al., 2023). According to our findings, wolfberry enhances the nuclear expression of YAP1 in hepatic cells. It has been shown that increased YAP1 expression promotes liver repair (Wang et al., 2022; Xie et al., 2021), while hepatic FXR expression has an inverse correlation with the degree of damage (Hartmann et al., 2018). Previous studies validated the association between YAP1 and FXR, demonstrating that FXR expression was significantly reduced in *Yap1* knockout mice, leading to downregulated CYP7A1 expression. Therefore, Thus, the CYP7A1 pathway mediated by FXR is possibly regulated by YAP1. In summary, wolfberry activated the YAP1/FXR pathway to reduce CYP7A1 expression and suppress excessive bile acid synthesis.

The liver and kidney protective effects of wolfberry are deeply rooted in traditional Chinese medicine theory. The *Compendium of Materia Medica* clearly states, “nourishes the kidneys, brightens the eyes,” and “long-term use strengthens tendons and bones,” revealing its connection to the liver and kidney meridians (Li and Wang, 2023). The combined hepatorenal protection of wolfberry presents substantial therapeutic potential from a clinical standpoint. This presented a method of mitigating liver and renal toxicity induced by antituberculosis medications.

## 5 Conclusion

Wolfberry comprehensively prevented liver damage under the condition of gut microbiota presence by enhancing gut microbiota diversity, strengthening intestinal barrier function, associating with the YAP1/FXR pathway.

## Data Availability

The 16S rRNA sequencing data have been deposited to the NCBI SRA, with the accession number PRJNA1348108: https://www.ncbi.nlm.nih.gov/bioproject/PRJNA1348108.
